# Strongyloides Hyperinfection Causing Gastrointestinal Bleeding and Bacteremia in an Immunocompromised Patient

**DOI:** 10.7759/cureus.15902

**Published:** 2021-06-24

**Authors:** Juan Carlos De la Cruz Mayhua, Bisharah Rizvi

**Affiliations:** 1 Internal Medicine, Saint Agnes Medical Center, Fresno, USA

**Keywords:** strongyloides hyperinfection, strongyloidiasis, immunocompromised patient, upper gastro-intestinal bleed, chronic steroids, gi bleed, strongyloides stercoralis, intestinal nematode

## Abstract

Strongyloidiasis is a parasitic infestation caused by *Strongyloides stercoralis *(*S. stercoralis).* Most cases are asymptomatic or mildly symptomatic with respiratory, gastrointestinal, or non-specific cutaneous symptoms. However, in immunocompromised patients, such as patients on chronic corticosteroids, malignancy, or human immunodeficiency virus (HIV) infection, hyperinfection syndrome can occur. The following is a case of *Strongyloides *hyperinfection in an individual taking prednisone for uveitis who developed upper gastrointestinal (GI) bleed and gram-negative bacteremia.

## Introduction

Strongyloidiasis is a parasitic infestation caused by the intestinal nematode, *Strongyloides stercoralis* (*S. stercoralis)*, that infects millions of individuals worldwide [1]. It is endemic in Southeast Asia, Latin America, Africa, and South Eastern United States. Most cases are asymptomatic or mildly symptomatic with respiratory, gastrointestinal (GI), or non-specific cutaneous symptoms [2]. However, in immunocompromised patients, such as patients with chronic corticosteroid use, malignancy, or human immunodeficiency virus (HIV) infection, hyperinfection syndrome or a disseminated disease can manifest. Recognizing strongyloidiasis early is paramount, as both morbidity and a high rate of mortality are linked with* S. stercoralis* hyperinfection or disseminated disease [3,4]. Hyperinfection syndrome is characterized by an acceleration of *S. stercoralis*' normal life cycle, resulting in an excessive worm burden inside the typical reproductive route (the skin, stomach, and lungs), while disseminated strongyloidiasis is characterized by extensive larval spread outside GI and respiratory organs, frequently affecting the heart, brain, and urinary tract [5]. The following is a case of an immunocompromised individual, where the duodenum was infected by* S. stercoralis* who presented with upper GI bleed and gram-negative bacteremia. 

## Case presentation

A 53-year-old male with past medical history of non-insulin-dependent diabetes mellitus, chronic alcoholism who quit drinking six months prior to admission, non-infectious uveitis diagnosed three months prior to presentation on oral prednisone, arrived with a three-month history of pain in his upper abdomen, which had worsened in the past one week. It was located in the epigastric region, described as pressure-like, and was 6/10 in intensity. Food intake made it worse, and nothing relieved it. It was associated with nausea and non-bloody emesis because of which he could not take his oral prednisone for a week. He denied any history of fever, diarrhea, bloody stool, or melena. He reported that all his symptoms started after starting 80 mg of prednisone daily for his uveitis. The patient worked in the fields harvesting fruits for over 10 years and was required to travel to different states. He immigrated from Mexico 25 years ago and his last visit was four years ago.

On presentation, he was alert but in moderate distress due to persistent hiccups. The vital signs showed fever of 39.2°C, blood pressure of 123/79, and pulse rate of 156 beats/minute with regular rhythm. On physical examination, he had tenderness over the epigastric region and right upper quadrant. Respiratory and cardiac examinations were normal. Initial laboratory findings showed hemoglobin of 11.7 g/dL, total white cell count of 5.5 x 10^9^/L without eosinophilia, and 26% bands. Basic metabolic panel showed hyponatremia (127 mmol/L), hypokalemia, hypomagnesemia, and normal renal function. Liver function profile was unremarkable. Blood cultures were positive for gram-negative bacilli in all four bottles. Computed tomography of the abdomen/pelvis done in the emergency department showed cholelithiasis with dilated common bile duct to 11 mm. Initially, a diagnosis of cholecystitis with gram-negative bacteremia was made. He was started on intravenous (IV) piperacillin-tazobactam. A hepatobiliary iminodiacetic acid (HIDA) scan was negative for acute cholecystitis. A gastroenterologist was consulted who performed a magnetic resonance cholangiopancreatography (MRCP) that showed a contracted gallbladder with gallstones, without stones in common bile duct stone.

**Figure 1:**
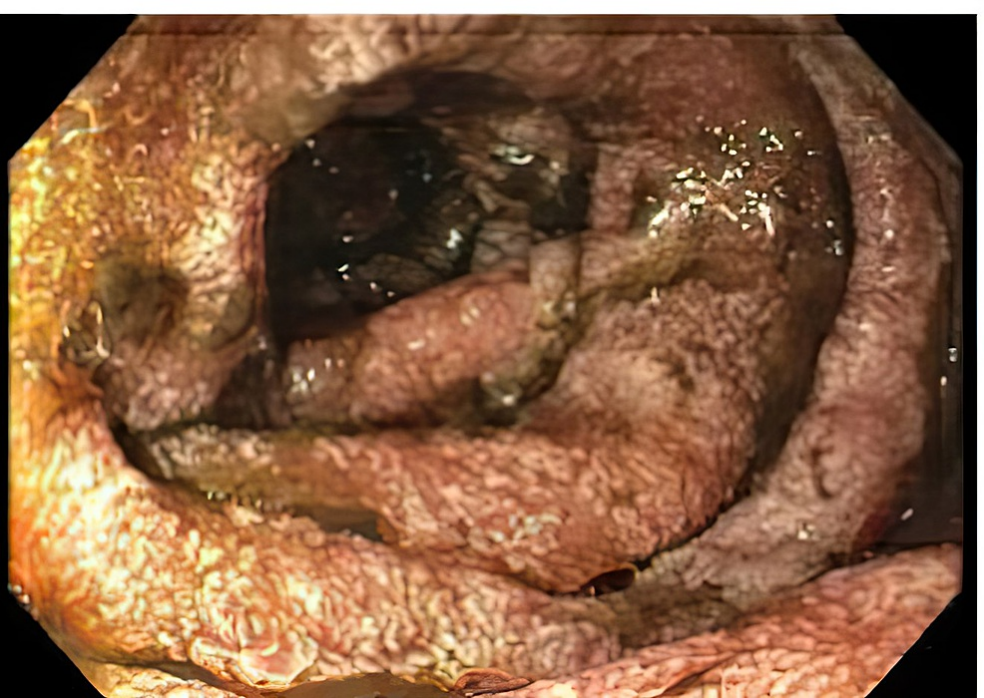
Diffuse moderate inflammation characterized with erythema, friability, and granularity in the second portion of the duodenum.

**Figure 2:**
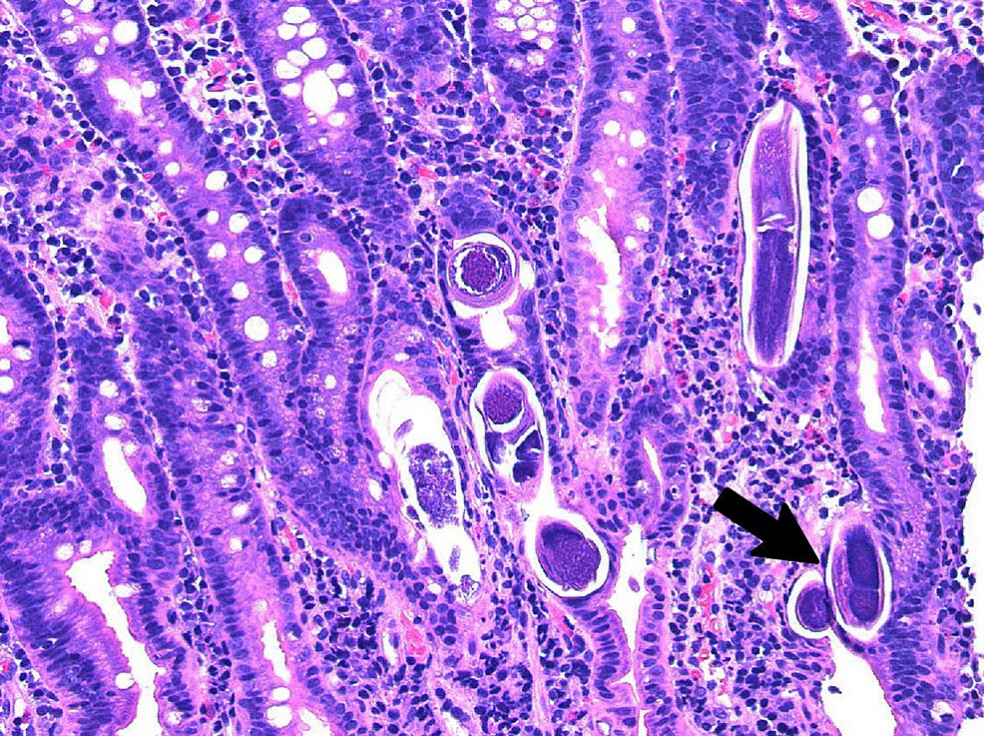
Haematoxylin and eosin (H&E) stain, duodenal mucosa x200 showing cross section (arrow) of rhabditiform larvae of Strongyloides stercoralis identified within the crypts of the duodenum. Inflammatory scattered cell infiltrate is also showed in the interstitial stroma.

**Figure 3:**
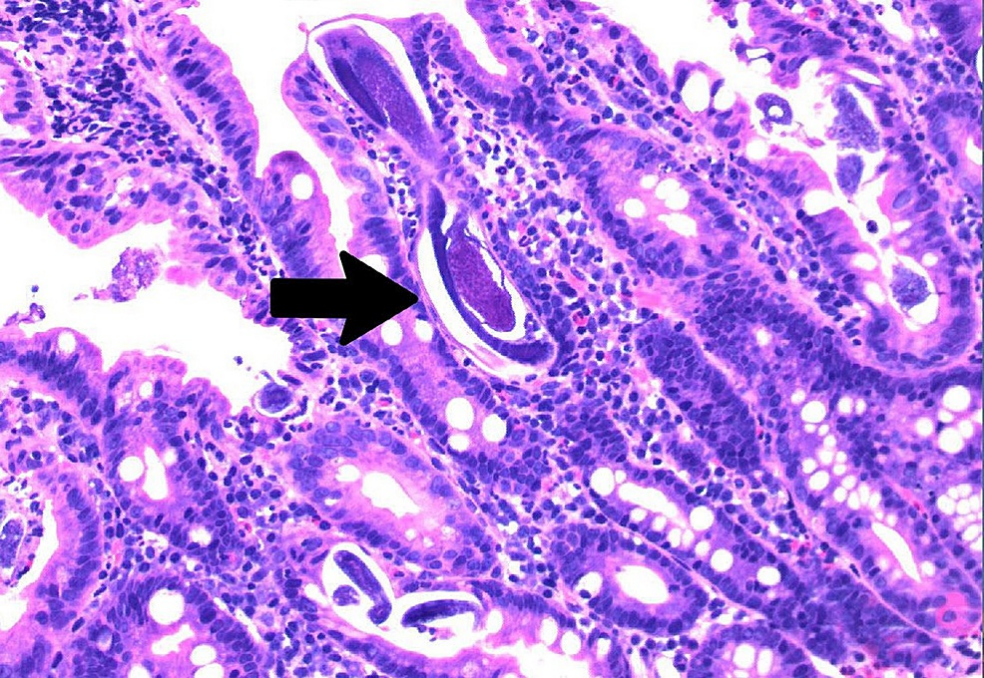
Haematoxylin and eosin (H&E) stain, duodenal mucosa x200 showing longitudinal section (arrow) of rhabditiform larvae of Strongyloides stercoralis.

On the second day after admission, the patient developed two episodes of coffee-ground emesis and was noted to have melena. His hemoglobin dropped from 13.5 g/dL to 9.8 g/dL. White cell count was 5.0 x 10^9^/L with eosinophilia at 9%. Intravenous pantoprazole was started. Due to upper GI bleed, he underwent an esophagogastroduodenoscopy (EGD) that showed LA grade D reflux esophagitis, nodular mucosa in the entire stomach, and duodenitis (Figure 1). Pathology of distal esophageal biopsy showed erosive esophagitis with granulation tissue; gastric biopsy showed superficial gastritis with minimal atrophy, negative for *Helicobacter pylori*; all four duodenal biopsies showed focal active duodenitis with strongyloidiasis. *S. stercoralis* rhabditiform larvae located within the crypts of the duodenum, along with inflammatory cells such as eosinophils, were identified on pathology slides (Figures 2-3).

Stool studies were negative for *Strongyloides*. The patient was treated with two days of oral ivermectin 15 mg. Blood culture collected on the day of admission came positive for *Klebsiella pneumoniae*, which was treated with ceftriaxone 2 g IV daily for two weeks. While in the hospital, the patient had persistent abdominal pain, nausea, non-bloody emesis, tachycardia, and an overall slow recovery process. The patient underwent a second EGD 12 days after presentation; pathology of duodenal biopsies showed duodenal mucosa with focal villous atrophy, elongation of crypts, and mild eosinophilia with no strongyloidiasis. On the 14th day of hospital admission, the patient was asymptomatic and hemodynamically stable and therefore was discharged with pantoprazole 40 mg twice daily. Additionally, the patient was seen in the outpatient clinic two weeks later without a relapse of symptoms. The steroid dosage was gradually tapered down over a one-month span.

## Discussion

Strongyloidiasis is endemic in tropical and subtropical regions of the world and has a prevalence of approximately 4% in the Southeastern region of the U.S [1]. The highest frequency of cases is seen in immigrants, travelers, and military personnel from endemic regions. *S. stercoralis*’ infectious cycle can be split into three segments. First is the direct cycle, which starts when the rhabditiform larvae from stool gives rise to filariform larvae which later enters through the skin. From the skin, larvae can travel through the bloodstream, where it can enter the lungs, resulting in the host possibly manifesting respiratory symptoms. The worms are ingested and reach the gastrointestinal tract through coughing. The larvae grow into egg-producing females in the small intestine, and these eggs develop into rhabditiform larvae. In the second cycle (indirect cycle), through the maturation of eggs, living adults mature in the soil. And in the third (the autoinfection cycle), matured rhabditiform formed from filariform larvae enter perianal skin and continue the cycle [6-10]. 

Depending on the acuity of infection and the host’s underlying immune response, the manifestations of strongyloidiasis vary case by case. A large majority of patients with strongyloidiasis live asymptomatically and continue for decades undiagnosed [11]. If symptomatic, strongyloidiasis can present with nausea, vomiting, diarrhea, abdominal pain, weight loss, GI bleed, cough, fever, and shortness of breath. Hyperinfection and disseminated disease have similar symptoms but because of increased parasite turnaround and dissemination, catastrophic complications can occur like shock, disseminated intravascular coagulation, GI hemorrhage, pulmonary hemorrhage, respiratory failure, or renal failure. Few case reports have been reported in which hyperinfection occurs due to the use of corticosteroids [12-17]. 

*S. stercoralis* has steroid receptors, which may have a role in the pathophysiology of hyperinfection syndrome and more systemic disseminated infection caused by corticosteroids, according to one study, although more research is needed in this area [18]. This was the case in our patient, his symptoms only manifested after becoming immunosuppressed from the high doses of corticosteroids for the treatment of noninfectious uveitis. Normal GI bacteria is carried into the bloodstream by worms moving across the intestinal mucosa. This mechanism happens in disseminated infection, or hyperinfection, and may result in gram-negative bacteremia [19], which was the case in our patient, who was found to have *Klebsiella pneumoniae *bacteremia. Higher mortality rates in hyperinfection or disseminated disease are usually due to secondary bacterial infections [20]. *S. stercoralis *infection should be suspected if an individual presents with GI symptoms, in conjunction with eosinophilia [6,19]. Due to steroid use, our patient did not have eosinophilia at first since steroid use is known to suppress the eosinophil count in blood [21]. 

The easiest way to diagnose strongyloidiasis is recognition of larvae in body samples, such as feces, sputum, or bronchoalveolar secretions. Up to 70% of cases have a negative single stool examination; and furthermore, when the same stool sample is examined three times, achieving a positive result is only 46% [6,9,22,23]. In our case, after two samples the stool analysis remained unrevealing. Other methods of detecting and diagnosing include culture of strongyloidiasis and serological methods, as well as stool concentration techniques. Though the enzyme-linked immunosorbent assay (ELISA) is a supplementary option, it does not distinguish between previous infection and other helminthic infections, nor is it always accessible [6,24]. In addition, the test’s sensitivity may be lower in severely immunocompromised patients, making a definitive diagnosis difficult [25-27]. Endoscopy-guided biopsies can help with the diagnosis of strongyloidiasis. Endoscopy is the most sensitive diagnostic procedure for diagnosing strongyloidiasis; the chances of obtaining a false negative result are less than 10% [4]. In our patient, the diagnosis was made by endoscopic biopsy of GI mucosa. Early diagnosis and treatment of* Strongyloides* hyperinfection is imperative, as the infection’s mortality rate is 50% in post-transplant patients and is as high as 87% in patients with hyperinfection from fatal GI bleed, respiratory distress, or septic shock [26]. First-line treatment is ivermectin, 200 ug/kg for two days [6,27]. Alternative options are albendazole, thiabendazole, or mebendazole [6].

## Conclusions

In conclusion, we suggest a high degree of clinical suspicion in specific populations where endemic *S. stercoralis* is present. Individuals who display symptoms of nausea, unintentional weight loss, and fatigue should have a detailed history taken to generate early diagnosis and deter development of complications, such as hyperinfection, especially in immunocompromised patients. Obtaining a thorough social history, such as work, and travel history is crucial. This case highlights the significance of keeping strongyloidiasis in the differential of high-risk patients presenting with acute GI symptoms. Early diagnosis and treatment of *Strongyloides *hyperinfection is imperative, as the infection’s mortality rate is high in immunocompromised individuals. 
